# Body mass index and type 2 diabetes in Thai adults: defining risk thresholds and population impacts

**DOI:** 10.1186/s12889-017-4708-7

**Published:** 2017-09-15

**Authors:** Keren Papier, Catherine D’Este, Chris Bain, Cathy Banwell, Sam-ang Seubsman, Adrian Sleigh, Susan Jordan

**Affiliations:** 1 National Centre for Epidemiology and Population Health (NCEPH) and Department of Global Health, Research School of Population Health, College of Medicine, Biology and Environment, The Australian National University, Canberra, Australia; 2 Population Health Department, QIMR Berghofer Medical Research Institute, Brisbane, Australia; 3 Thai Health-Risk Transition Study, School of Human Ecology, Sukhothai Thammathirat Open University, Nonthaburi, Thailand; 4 The School of Public Health, The University of Queensland, Brisbane, Australia; 5 National Centre for Epidemiology & Population Health, The Australian National University, Building 62, Mills Road, Canberra, ACT 2601 Australia

**Keywords:** Body mass index, Diabetes, Cut-points, Population attributable fraction, Asian cohort

## Abstract

**Background:**

Body mass index (BMI) cut-off values (>25 and >30) that predict diabetes risk have been well validated in Caucasian populations but less so in Asian populations. We aimed to determine the BMI threshold associated with increased type 2 diabetes (T2DM) risk and to calculate the proportion of T2DM cases attributable to overweight and obesity in the Thai population.

**Methods:**

Participants were those from the Thai Cohort Study who were diabetes-free in 2005 and were followed-up in 2009 and 2013 (*n* = 39,021). We used multivariable logistic regression to estimate odds ratios (ORs) and 95% confidence intervals (CIs) for the BMI-T2DM association. We modelled non-linear associations using restricted cubic splines. We estimated population attributable fractions (PAF) and the number of T2DM incident cases attributed to overweight and obesity. We also calculated the impact of reducing the prevalence of overweight and obesity on T2DM incidence in the Thai population.

**Results:**

Non-linear modelling indicated that the points of inflection where the BMI-T2DM association became statistically significant compared to a reference of 20.00 kg/m^2^ were 21.60 (OR = 1.27, 95% CI 1.00–1.61) and 20.03 (OR = 1.02, 95% CI 1.02–1.03) for men and women, respectively. Approximately two-thirds of T2DM cases in Thai adults could be attributed to overweight and obesity. Annually, if prevalent obesity was 5% lower, ~13,000 cases of T2DM might be prevented in the Thai population.

**Conclusions:**

A BMI cut-point of 22 kg/m^2^, one point lower than the current 23 kg/m^2^, would be justified for defining T2DM risk in Thai adults. Lowering obesity prevalence would greatly reduce T2DM incidence.

**Electronic supplementary material:**

The online version of this article (10.1186/s12889-017-4708-7) contains supplementary material, which is available to authorized users.

## Background

In recent decades, many low and middle-income countries (LMICs) have achieved substantial economic growth and this has led to increased urbanization, the adoption of new health behaviours (e.g. smoking and a ‘western’ diet), and an epidemiological shift from infectious diseases towards non-communicable diseases including type 2 diabetes mellitus (T2DM). These changes in health-risk behaviours, environment, and health outcomes, termed the ‘health-risk transition’, have been occurring in Thailand and T2DM now affects over four million adults [1].

Overweight and obesity significantly increase the risk of T2DM [2, 3] and their prevalence is increasing in Thailand [4]. Of concern, the association between body mass index (BMI) and T2DM risk is modified by ethnicity, with Asian populations having an increased risk of T2DM at BMI levels considered to be in the healthy weight range for Caucasian populations (<25 kg/m^2^) [5]. Accordingly, in 2000 the World Health Organization (WHO) recommended that lower BMI cut-off points should be used to define overweight (23- < 25 kg/m^2^) and obesity (>25 kg/m^2^) in Asian populations [6]. These recommendations were based on the few prevalence studies at the time [7–10]. In 2004, the WHO released an additional statement indicating that a range of BMI cut-off points may be necessary for guiding public health action in different Asian populations since the available data were inconsistent [11].

Since publication of these two recommendations, many studies assessed the validity of lower Asian-specific BMI cut-points for assessing diabetes, cardiovascular and mortality risk. Results were inconclusive, potentially due to confounders or exclusion of women [12], cross-sectional design [5, 13–15] or small samples of Asian participants [5, 16]. The prevalence of overweight and obesity are projected to continue to rise in Thailand. Understanding the optimal Southeast Asian BMI cut-off values associated with T2DM risk and the contribution of excess weight to the development of T2DM will have major implications for public health planning in Thailand. Therefore we examined the BMI threshold associated with T2DM risk in a large prospective cohort of adult Thais. We also estimated population attributable fractions and number of T2DM cases attributable to overweight and obesity in Thailand.

## Methods

### Study population

The Thai Cohort Study (TCS), is a nation-wide prospective investigation of the evolving ‘health-risk transition’ in Thailand [17]. TCS members (*N* = 87,151) were distance-learning students enrolled at Sukhothai Thammithirat Open University (STOU) who completed the baseline questionnaire in 2005 (44% of the total STOU student body). Follow-up questionnaires sent out in 2009 and 2013 were completed by 70% (*n* = 60,569) and 49% (*n* = 42,785) respectively of the original baseline cohort. The questionnaires collected information on a wide-range of topics including socio-demographic, health and lifestyle factors, and health outcomes (including diabetes).

### Eligibility

Participants were eligible for this analysis if at baseline (2005) they reported not having diabetes and had a valid BMI (greater than 12.0), and provided a diabetes status in 2013.

### T2DM Statu**s**

Participants were classified as having diabetes if they responded positively to the question “Have you ever received a confirmed diagnosis from a doctor that you definitely have diabetes?” by 2013. A validation study of self-reported diabetes among TCS participants, undertaken by a practicing Thai physician, indicated that the accuracy of diabetes self-report was high (82%), particularly among those who reported doctor-diagnosed diabetes in both 2009 and 2013 (96%) [18].

### Body mass index

BMI was calculated using self-reported weight and height at baseline (Weight (kg)/ Height (meters^2^)). A validation study indicated that these measures were accurate and reliable [19]. BMI was analysed as both a continuous and categorical variable. For the categorical variable, we created 8 categories (<18.5, 18.5 ≥ to <20.75, 20.75 ≥ to <23, 23 ≥ to <25, 25 ≥ to <27.5, 27.5 ≥ to <30, ≥ 30 to < 32.5, and ≥32.5) based on the 2000 International Task Force (ITF) [6] and the 2004 WHO [11] recommendations. To allow for finer grading of T2DM risk at lower BMI levels, we created two additional categories between 18.5 and 23.0 [5]. We combined the two highest categories into one because these groups were small.

### Covariates

Potential confounders from the baseline questionnaire included socio-economic characteristics (income (<10,000 Baht per month, 10,001–20,000 Baht per month, >20,001 Baht per month) and education level (Junior high school, High school, Diploma/Certificate, University)); demographic factors (age and childhood area of residence (urban/rural)); lifestyle factors (smoking (never smoked, ex-smoker, current smoker) and alcohol consumption (never, ex-drinker, occasional/social drinker, regular drinker)); fruit and vegetable consumption (categorised as < five or ≥ five serves/day), and consumption of sugar sweetened beverages (SSBs) (<3×/month, 1–6/week, 1+/day). Leisure physical activity, reported as number of sessions per week of strenuous, moderate or mild exercise, was weighted (“2 × strenuous + moderate + mild + walking” exercise sessions) [20] and categorized by sessions per week (none, 1–7, 8–14, 15 or more) [21].

### Statistical analysis

Since the relationship between diabetes and BMI may differ by sex, we conducted all analyses separately for men and women [22]. For eligible participants, baseline characteristics were compared for those with and without T2DM in 2013.

We used multivariable logistic regressions to assess the association between baseline BMI categories and development of T2DM by 2013. In Model 1 we estimated age-adjusted odds ratios (OR) and 95% confidence intervals (CI) for each BMI category (unadjusted for other variables). We then added potential confounders of the BMI-T2DM association (Model 2). These variables were identified using directed acyclic graphs (DAGs) based on theoretical knowledge and previous work with this cohort. They included age, area of residence during childhood, education, income, physical activity, consumption of fruit/vegetables, sugar sweetened beverage intake [23], alcohol [24], and smoking [25].

We also modelled non-linear associations between baseline BMI (using a continuous term) and T2DM risk using restricted cubic splines with sex-specific distributions for BMI (using four knots, at 5th, 35th, 65th and 95th percentiles) [26]. To test for non-linearity, we compared one model with the linear BMI term to another model including the linear BMI term and its splined terms using a Wald test for men and women, respectively. We then determined the point of inflection as the lowest BMI value for which the association between BMI and T2DM was statistically significant using a BMI reference point of 20.00 and an increment of 0.01.

Since previous work with other Asian cohorts and Thai adults suggests that the relationship between BMI and T2DM risk could vary by age and urbanization status [11, 27] we stratified the models by baseline age (under 30, 30–39, and 40 or over) and childhood area of residence (rural versus urban). We also added the interaction terms of interest (categorical BMI x age or urbanization status x categorical BMI) to the main regression model (Model 3).

### Proportion of T2DM cases attributable to overweight and obesity

We calculated population attributable fractions (PAFs) of overweight and obesity for each age-sex group using the standard formula [28] $PAF\%=\frac{\sum \left( px\times \left( OR-1\right)x\right)}{1+\sum \left( px\times \left( OR-1\right)x\right)}\times 100$

*where px is the proportion of the population in the exposure level x (separate for overweight and obesity, categorized using the criteria of 23- < 25 kg/m* ^*2*^ *for overweight and* *>* *25 for obesity for comparability with previous studies) and OR-1 is the excess risk associated with exposure level x)* to determine the proportion of T2DM in the cohort that could have been prevented if participants had a BMI of <23 kg/m^2^. We then applied the sex-specific eight-year cumulative incidence and PAFs from this study to the total number of men and women in the national Thai population and divided the results by eight to estimate the annual number of T2DM cases in the national Thai population that could be attributed to a BMI of >23 kg/m^2^ annually. As well, since we found the inflection points for BMI significantly associated with T2DM risk in TCS men and women were 21.60 and 20.03, we also calculated the effect of reducing BMI levels from <23 kg/m^2^ to <22 kg/m^2^ and from <23 kg/m^2^ to <21 kg/m^2^ in TCS men and women, respectively.

### Impact of a theoretical 5% reduction in the prevalence of overweight and obesity in the TCS

We estimated the potential impact fraction (PIF) [29] that a 5% reduction in the prevalence of obesity and of overweight in the TCS cohort could have on T2DM incidence. This hypothetical impact was modelled as follows: 1) we reduced the prevalence of obesity (>25 kg/m^2^) in the cohort to a level 5% below the original level and increased the prevalence of overweight (23- < 25 kg/m^2^) by 5%; 2) we reduced the prevalence of overweight in the cohort by 5% and increased the prevalence of normal weight (<23 kg/m^2^) by 5%. We calculated these PIFs using the formula [29] $PIF\%=\frac{\left(\left(\sum p\times OR\right)-\left(\sum {p}^{\ast}\times OR\right)\right)}{\left(\sum p\times OR\right)}\times 100$ *where p is the proportion of TCS members with overweight or obesity (categorized using the criteria of 23- < 25 kg/m* ^*2*^ *for overweight and* *>* 25 for obesity), OR is the odds of T2DM for each BMI category, and p* is an absolute 5% reduction in the real proportion of TCS members with overweight or obesity.

Using the PIFs we calculated the hypothetical sex-specific T2DM incidence that would have occurred in the TCS had the prevalence of overweight and obesity been 5% lower. We then applied the observed and resulting hypothetical sex-specific T2DM incidences from our cohort to the national Thai population to estimate the number of T2DM cases that could be prevented annually if the hypothetical reductions in the prevalence of overweight and obesity were achieved.

All analyses were carried out using Stata (version 13.0). All statistical tests were two-sided.

## Results

Of the 87,151 initial TCS participants, 60,569 were followed-up in 2009. Of these, 706 had prevalent diabetes in 2005 and 28 did not have a response for the diabetes question in 2009 and were excluded. Of the remaining 59,835, a total of 39,507 eligible participants (without missing diabetes responses) were followed-up in 2013. Of these, 486 did not have credible height or weight data. The final study sample included 39,021 participants of whom 688 reported a new diagnosis of diabetes (see flow chart in Additional file 1: Figure S1).

The baseline characteristics of participants by sex and diabetes status are shown in Table 1. Among both men and women, T2DM incidence increased with increasing age, BMI, and income (*p* < 0.001). T2DM incidence was double among those who lived in a city rather than a rural area as a child (*p* < 0.001). Among women, T2DM incidence was highest in those who consumed sugar-sweetened beverages (SSBs) daily (*p* < 0.01). Among men, T2DM incidence was highest in current smokers and those who consumed alcohol regularly (*p* < 0.001).

**Table 1:** Thai Cohort Study: baseline characteristics (2005) by diabetes outcome in 2013

	Men *N* = 17607^a^			Women *N* = 21900^a^		
	T2DM incidence^c^	%^d^	p ^e^	T2DM incidence ^c^	%^d^	p ^e^
*Total*	438/17607	2.5		260/21900	1.2	
Age years						
Under 30	61/7029	0.9	<0.001	60/12097	1.0	<0.001
30–39	166/6634	2.5		108/7101	1.5	
40 or over	211/3944	5.4		92/2702	3.4	
BMI-Asian (kg/m^2^) cut-points^b^						
Underweight (<18.49)	5/962	0.5	<0.001	3/4221	0.1	<0.001
Normal (18.5- < 23.0)	67/8208	0.8		56/12789	0.4	
At risk (23.0- < 25.0)	76/4034	1.9		45/ 2347	1.9	
Obese I (25.0- < 30.0)	196/3631	5.4		98/ 1858	5.3	
Obese II (>30.0)	86/520	16.5		56/ 451	12.4	
Income (Baht/month)						
< 10,000	146/ 8930	1.6	<0.001	121/ 13,996	0.9	<0.001
10,001–20,000	148/ 5590	2.7		79/ 5338	1.5	
> 20,001	134/ 2820	4.8		54/ 2087	2.6	
Education level						
Junior high school	21/ 769	2.7	0.16	6/ 376	1.6	0.90
High school	177/ 7991	2.2		94/ 7948	1.2	
Diploma/certificate	101/ 3994	2.5		79/ 6670	1.2	
University	137/ 4815	2.8		79/ 6849	1.2	
Childhood area of residence						
Rural	290/13377	2.2	<0.001	158/ 15,871	1.0	<0.001
Urban	141/ 4045	3.5		96/ 5846	1.6	
Fruit and vegetable serves/day						
< 5 serves	310/ 11,658	2.7	0.05	156/ 12,619	1.2	0.37
> 5 serves	115/ 5360	2.2		95/ 8625	1.1	
Sugar sweetened beverage intake						
Less than daily	404/ 16,376	2.5	0.24	230/ 20,424	1.1	<0.01
> daily	33/ 1083	3.0		28/ 1292	2.2	
Smoking						
Never smoked	147/ 8342	1.8	<0.001	237/ 20,290	1.2	0.17
Ex-smoker	151/ 5437	2.8		6/ 691	0.9	
Current smoker	115/ 3024	3.8		4/ 149	2.7	
Alcohol intake						
Never drinks	29/ 1868	1.6	<0.001	123/ 9005	1.4	0.14
Quit	59/ 1777	3.3		12/ 1475	0.8	
Occasional drinker	280/ 12,037	2.3		119/ 11,012	1.1	
Regular drinker	67/ 1780	3.8		1/ 120	0.8	

^a^Numbers may not add to total sample size due to missing responses for some characteristics

^b^Body mass Index (BMI) defined using the WHO International Obesity Taskforce recommendations

^c^Incident cases cumulating by 2013 divided by population without diabetes at baseline

^d^Cumulative incidence over 8 years T2DM, type 2 diabetes mellitus

^e^Chi Square *p* value comparing baseline characteristics among participants by T2DM status in 2013

**Fig. 1:**
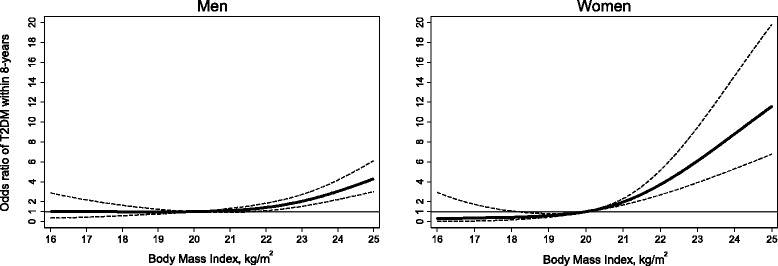
Odds ratios of type 2 diabetes incidence (2005–2013) according to body mass index in 2005. - - - - 95% Confidence Interval T2DM risk modelled using restricted cubic splines with knots at 5th, 35th, 65th and 95th. Body mass index reference was set at 20.00 kg/m^2^

The age and multivariable adjusted sex-specific associations between baseline BMI and T2DM incidence by 2013 are shown in Table 2 and in Fig. 1. A BMI of 20.75- < 23.00 (compared to 18.5- < 20.75) was associated with higher T2DM risk in women (OR =3.0, 95% CI 1.6–5.7). In men, a BMI of 23 < 25 (compared to 18.5- < 20.75) was associated with higher T2DM risk (OR = 2.3, 95% CI 1.3–3.9). The cubic spline non-linear modelling of BMI’s effect on T2DM showed that the points of inflection where the association became statistically significant compared to a reference of 20.00 kg/m^2^ for men and women were 21.60 (OR = 1.27, 95% CI 1.00–1.61) and 20.03 (OR = 1.02, 95% CI 1.02–1.03), respectively. In women, BMI levels <20 kg/m^2^ appeared to be inversely associated with risk suggesting thresholds might be even lower for women, however, few participants fell into these categories thus definite conclusions are difficult to draw. At a BMI of 25.0 kg/m^2^, T2DM risk was exponentially increased in both men (OR = 4.3, 95% CI 3.0–6.1) and women (OR = 11.6, 95% CI 6.8–19.8.), respectively. These findings suggest that the exponential relationship between BMI and T2DM is greater among women than men and that excess adiposity in Southeast Asian women has major implications for T2DM risk even at BMI levels considered to be in the healthy weight range for Asian populations (<23 kg/m^2^) (Fig. 1).

**Table 2:** Association between baseline body mass index and eight-year incidence of type 2 diabetes

Body Mass Index (kg/m^2^) at baseline in 2005	Incident cases by 2013^a^	Adjusted OR estimates relating BMI and T2DM	
		Model 1^b^ OR (95% CI)	Model 2^c^ OR (95% CI)
Men			
< 18.5	5/968	0.94 (0.34–2.5)	0.85 (0.28–2.6)
18.5 - <20.75	19/3232	1	1
20.75 - <23.0	48/4970	1.4 (0.80–2.3)	1.2 (0.69–2.2)
23.00 - < 25.00	77/ 4054	2.3 (1.4–3.8)	2.3 (1.3–3.9)
25.00 - <27.50	120/ 2583	5.5 (3.4–9.0)	5.5 (3.2–9.2)
27.50 - <30.00	75/1028	8.6 (5.1–14.4)	8.1 (4.7–14.1)
30.00 - <32.50	52/369	20.2 (11.7–34.8)	22.6 (12.7–40.1)
32.5 and over	34/151	42.3 (23.3–77.1)	43.3 (22.9–81.6)
Women			
< 18.5	3/4222	0.35 (0.10–1.2)	0.44 (0.12–1.5)
18.5 - <20.75	18/7602	1	1
20.75 - <23.0	38/5186	2.7 (1.5–4.7)	3.0 (1.6–5.7)
23.00 - <25.00	45/2373	6.1 (3.5–10.6)	6.7 (3.6–12.4)
25.00 - <27.50	63/1292	16.4 (9.6–27.9)	14.9 (8.1–27.5)
27.50 - <30.00	35/543	21.6 (12.0–38.7)	23.0 (12.0–44.1)
30.00 - <32.50	23/265	31.1 (16.4–58.8)	34.9 (17.4–69.7)
32.5 and over	33/183	78.6 (43.1–143.4)	73.5 (37.0–146.2)

*ORs* Odds ratios, *CI* Confidence Interval, *BMI* Body Mass Index in kg/m^2^ T2DM, type 2 diabetes mellitus

^a^Incident cases cumulating by 2013 divided by population without diabetes at baseline

^b^Model 1 is age adjusted

^c^Model 2 is adjusted for age, education, income, area of childhood residence, physical activity, smoking, alcohol intake, fruit and vegetable intake, and sugar sweetened beverage intake. These variables were selected using directed acyclic graphs (see methods)

**Fig. 2:**
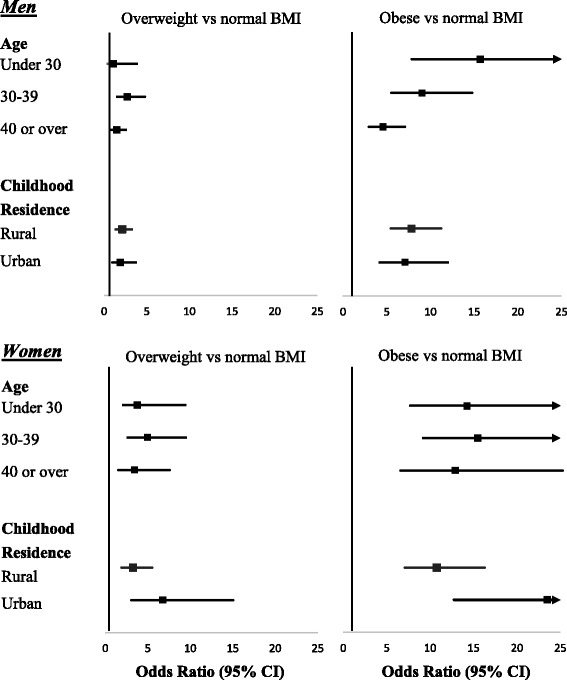
Odds ratios of type 2 diabetes by body mass index stratified by age and residence CI confidence interval Overweight: Body Mass Index (BMI) 23.00 - < =24.99 kg/m^2^ and Obesity BMI >25.00 kg/m^2^ Adjusted for age, education, income, area of childhood residence, physical activity, smoking, alcohol intake, fruit and vegetable intake, and sugar sweetened beverage intake

Figure 2 shows the sex-specific association between baseline BMI and eight-year T2DM incidence stratified by age and urbanization status. Among men, while the association between obesity (BMI > 25) and risk of T2DM was higher among participants aged <30 (OR = 15.7, 95% CI 7.9–31.4) than those aged 40 and older (OR = 4.60, 95% CI 2.98–7.11) the interaction term between BMI and age was not statistically significant (*p* = 0.47). Among women, there was some indication that the BMI-T2DM association was modified by area of residence as a child such that the association between high BMI categories (> 23 and >25) and T2DM risk was higher in participants who lived in an urban area of residence as a child (BMI > 23: OR = 6.8, 95% CI 3.1–15.0; > 25 OR = 24.6, 95% CI 12.7–47.7) than those who lived in a rural area of residence as a child BMI > 23: OR = 3.3, 95% 1.9–5.5; BMI > 25: OR = 10.8, 95% 7.1–16.3), however, the interaction term between BMI and urbanization status was not statistically significant at the 5% level (*p* = 0.08).

### Proportion of T2DM cases attributable to overweight and obesity

We estimated that around 63% of T2DM in men and 62% in women could be attributed to overweight or obesity (Table 3). Assuming the association between BMI and T2DM is causal, an estimated 66,000 cases in men and 32,000 in women per year may have been prevented in the national Thai population if a BMI level of <23 kg/m^2^ was maintained across the population. Moreover, reducing BMI levels from <23 kg/m^2^ to <22 kg/m^2^ and from <23 kg/m^2^ to <21 kg/m^2^ in TCS men and women, respectively would have prevented a further 6700 cases in men and 9500 cases in women annually, in the national Thai population.

**Table 3:** Thai Cohort Study: population attributable fraction^d^ of diabetes due to excess weight

	Overweight			Obese			Total^b^
Age group	Percent^a^	Odds Ratio^c^ (95% CI)	PAF%	Percent^a^	Odds Ratio^c^ (95% CI)	PAF%	PAF %
Men							
< 30	16.0	1.10 (0.30–3.96)	0.5	13.9	15.73 (7.87–31.4)	66.8	67.3
30–39	26.8	2.72 (1.54–4.80)	12.5	27.5	9.06 (5.55–14.78)	60.3	72.8
> 40	28.2	1.51 (0.89–2.56)	5.8	36.9	4.60 (2.98–7.11)	53.8	59.6
Total	21.6	2.06 (1.44–2.96)	8.5	22.2	7.65 (5.69–10.28)	54.6	63.1
Women							
< 30	6.6	3.78 (1.50–9.52)	8.8	6.8	14.24 (7.70–26.3)	43.0	51.8
30–39	13.0	4.98 (2.62–9.48)	15.0	13.3	15.50 (9.19–26.2)	56.0	71.0
> 40	21.8	3.44 (1.56–7.58)	12.6	22.5	12.91 (6.59–25.27)	63.6	76.2
Total	9.8	4.13 (2.68–6.35)	11.6	9.8	14.23 (10.11–20.02)	50.0	61.6

*CI* confidence interval, *PAF* Population Attributable Fraction

Overweight: Body Mass Index (BMI) 23.00- < =24.99 kg/m^2^ and Obesity: BMI >25.00 kg/m^2^

^a^Prevalence

^b^Overweight and Obesity combined

^c^Odds ratios (ORs) associating baseline body mass index and eight-year incidence of type 2 diabetes between 2005 and 2013. All ORs are adjusted for age, education, income, area of childhood residence, physical activity, smoking, alcohol intake, fruit and vegetable intake, and sugar sweetened beverage intake

^d^ PAF% calculated using the formula $PAF\%=\frac{\sum \left( px\times \left( OR-1\right)x\right)}{1+\sum \left( px\times \left( OR-1\right)x\right)}\times 100$

### Potential impact of reducing the prevalence of overweight and obesity in the TCS

Our results suggest that if the prevalence of overweight could be reduced by 5% then this could result in a slight reduction in T2DM cases annually in the national Thai population (male PIF 2.0%, cases prevented per year = 1300; female PIF 6.0%, cases prevented per year = 1900). A 5% reduction in the prevalence of obesity would have a more profound effect, potentially preventing ~6600 cases in men (PIF 10%) and ~6100 in women (PIF 19%) in the national Thai population.

## Discussion

Our prospective nationwide study of Thai adults provides further evidence that T2DM risk is increased at BMI levels below 25 kg/m^2^ in Asian populations and that the use of lower Asia-specific BMI cut-points are necessary for defining T2DM risk in Asian adults, particularly in women. We found that T2DM risk was associated with a BMI of 22 kg/m^2^ and 20 kg/m^2^ in men and women, respectively. We also found that for the same BMI level the association between BMI and T2DM risk was higher in women and that their T2DM risk was already increased at BMI levels currently considered in the ‘healthy range’ for Caucasian and Asian populations. Over 60% of all T2DM cases occurring in this cohort could be attributed to overweight and obesity and our results suggest that a hypothetical 5% reduction in the prevalence of obesity could result in almost 13,000 fewer cases of T2DM annually in the national Thai population.

There are several potential limitations which should be considered when interpreting our findings. All data on height, weight and diabetes diagnoses were ascertained using self-report. Accordingly, there may be some misclassification error which should be considered when interpreting these findings. However, validation studies of self-reported diabetes [18] and self-reported weight and height [30] in this cohort have shown that the accuracy of these self-reported measures is high. Another potential issue is attrition. Over the eight-year follow-up, 50% of the baseline cohort was lost to follow-up. Slight differential attrition was noted by body size with a higher retention rate for participants with a larger weight at baseline. However, previous work with this cohort showed that ORs for the association between BMI and T2DM incidence in the first 4 years (70% retention of baseline cohort) were similar to those from the total eight-year follow-up, indicating that eight-year ORs are likely to be generalizable [21]. Moreover, non-differential attrition was noted by sex, dietary intake, alcohol intake, and area of residence indicating that these variables would unlikely affect BMI-T2DM risk effect estimates (see Additional file 2: Table S1).

An additional issue to consider is the precision of the PAF and PIF estimates. These measures are dependent on the accuracy and the magnitude of the ORs and the prevalence estimates being used. Accordingly, there is potential for error in these estimates due to variations in the accuracy in the self-report of weight and height, differences in the prevalence of overweight and obesity between our cohort and the national Thai population, as well as any risk estimates affected by attrition in this cohort. There is no agreed method for calculating confidence intervals on composite measures such as PAF and PIF estimates. Accordingly, these were not calculated. Nevertheless, our PIF and PAF estimates highlight the potential magnitude of the effects of overweight and obesity on T2DM incidence in Thai adults.

Important strengths of this study include the large sample of Thai adults and our nationwide coverage. Moreover, to our knowledge this is the largest prospective study to assess the BMI cut-points associated with T2DM risk and the number of T2DM cases attributed to overweight and obesity in adults living in Southeast Asia.

This study found that T2DM risk is increased at BMI levels considered to be in the ‘normal’ range for Caucasian populations (<25 kg/m^2^). Our findings are consistent with previous studies that recommend using a BMI cut-off between 21 and 24 kg/m^2^ to define overweight and obesity in Asian populations based on diabetes and cardiovascular risk [5, 13–15, 31–33]. However, studies of body size and mortality could shed light on appropriate cut-offs for BMI on health more generally. For example, a few mortality studies conducted in different Asian populations did not find evidence that mortality risk was increased at BMI levels <25 kg/m^2^ [34]. However, these studies were unable to adjust for potential confounding effects of factors such as infectious diseases and the prevalence of smoking in these different populations. Adjusting for related factors in this study had minimal influence on the BMI-T2DM relationship. However, these factors might modulate more of the BMI-mortality association than the BMI-T2DM relationship [35].

A likely explanation for the increased risk of T2DM at these low levels of BMI is body composition. Evidence suggests that the relationship between BMI and body fat differs by ethnicity, with Asian populations having higher proportions of body fat compared to muscle mass than Caucasian populations when matched by age and BMI [36]. Ethnicity related differences in body fat composition and distribution may be associated with epigenetic programming [37]. Babies that develop in an undernourished intrauterine environment have been shown to experience ‘catch up’ growth later in life that has been associated with preservation of adipose tissue, obesity, and increased insulin resistance [38]. As well, children growing up with under-nutrition and frequent infection who survive to adulthood have low attained height, and a life-long threat of adult obesity, which is widespread in Thailand today [39]. Many Asian populations have experienced generations of under-nutrition prior to experiencing the accelerated nutrition transition currently underway in much of Asia. Accordingly, some Asian populations may be more predisposed to storing visceral fat or are more insulin resistant at lower levels of adiposity [36, 40].

Effects of epigenetic programming may be particularly intensified when there is a strong mismatch between the intrauterine and early life environments [41], which may explain the potentially increased association between obesity and T2DM incidence noted in TCS females who lived in an urban area of residence during childhood. Living in an urban area during childhood has been shown to be associated with increased exposure to an energy-dense western diet, reduced physical activity and a higher prevalence of obesity during adulthood [42]. Therefore, unlike children who are raised in a rural area of residence and may have low exposure to the ‘western diet’, those who are raised in an urban area of residence may have pro-longed exposure to an obesogenic environment and consequently an increased risk of T2DM later in life.

As in previous studies, this study found that for the same BMI T2DM risk was higher in women [22]. Evidence suggests that this sex-specific association may be related to differences in body composition and hormones between men and women. For instance, men have higher concentrations of testosterone than women and testosterone has been shown to inversely associate with adiposity and insulin resistance in men [43]. This effect may relate to testosterone’s role in increasing lean body mass and decreasing inflammatory cytokines, which can increase insulin resistance [43]. These findings highlight the need for public health interventions to target the promotion of healthy weight differently in Asian men and women.

Our findings show that the public health impact of preventing or reducing the prevalence of obesity in Thailand could be profound. Shifting as little as 5% of the population with obesity to a lower BMI of 23- < 25 kg/m^2^ could lead to a substantial decrease in the number of T2DM cases occurring in the Thai population; and this benefit could be even greater than we have calculated considering the high and increasing prevalence of obesity, particularly in women, in the national Thai population [27].

## Conclusions

The findings from this prospective study of Thai adults suggest that using lower BMI cut-points is necessary for defining T2DM risk in Southeast Asian populations. Our findings suggest that T2DM risk is already increased at BMI levels <23 kg/m^2^. Therefore, public health action and response may be required at lower BMI levels to help curb the T2DM epidemic currently underway in Southeast Asia. Further research is required to confirm these findings in different Southeast Asian populations.

## Additional files

Additional file 1: Figure S1. Selection of the analysed cohort from the Thai Cohort Study. (PDF 345 kb)

Additional file 2: Table S1. Baseline characteristics for participants versus non-participants. (DOCX 14 kb)

## Abbreviations

BMI: Body mass index
CI: Confidence intervals
DAG: Directed acyclic graph
ITF: International Task Force
LMICs: Low and middle-income countries
OR: Odds ratios
PAF: Population attributable fraction
PIF: Potential impact fraction
SSBs: Sugar sweetened beverages
STOU: Sukhothai Thammithirat Open University
T2DM: Type 2 diabetes mellitus
WHO: World Health Organization
