# Supplementary figures and images for: Body mass index and type 2 diabetes in Thai adults: defining risk thresholds and population impacts
Source: BMC Public Health. 2017 Sep 15;17:707. doi: 10.1186/s12889-017-4708-7 (PMC5602842; doi:10.1186/s12889-017-4708-7)

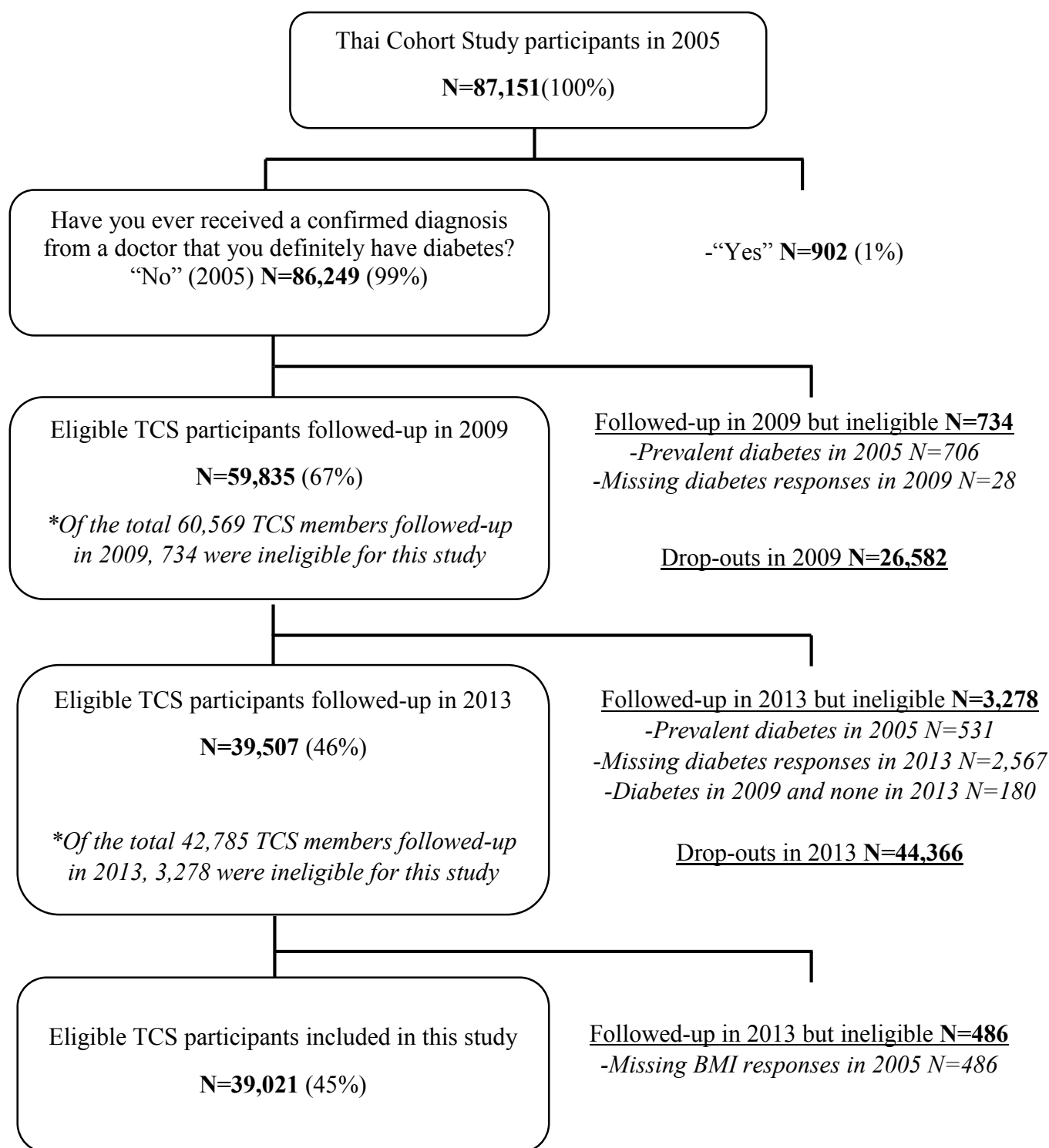

#### Additional file 1

**Figure: Selection of the analysed cohort from the Thai Cohort Study**

Supplement: Supplementary file 1 — Selection of the analysed cohort from the Thai Cohort Study. (PDF 345 kb) [file 12889_2017_4708_MOESM1_ESM.pdf]
