# Supplementary material for: Body mass index and type 2 diabetes in Thai adults: defining risk thresholds and population impacts
Source: BMC Public Health. 2017 Sep 15;17:707. doi: 10.1186/s12889-017-4708-7 (PMC5602842; doi:10.1186/s12889-017-4708-7)
Supplement: Supplementary file 2 — Baseline characteristics for participants versus non-participants. (DOCX 14 kb) [file 12889_2017_4708_MOESM2_ESM.docx]

**Additional file 2**

**Table: Baseline characteristics for participants *versus* non-participants**

| **Baseline characteristics** | **Non participants***  **n (%)** | **Study participants***  **n (%)** | **P value**** |
| --- | --- | --- | --- |
|  |  |  |  |
| ***Total*** | 48,130 (55%) | 39,021 (45%) |  |
| **Sex** |  |  | <0.001 |
| Men | 22,129(46) | 17,355(44) |  |
| Women | 25,994(54) | 21,666(56) |  |
| **Age years** |  |  | <0.001 |
| Under 30 | 31,304(65) | 18,963(48) |  |
| 30-39 | 11,951(25) | 13,570(35) |  |
| 40 or over | 4,857(10) | 6,488(17) |  |
| **BMI-Asian cut-points†** |  |  | <0.001 |
| Underweight (<18.49 | 7,475(16) | 5,183(13) |  |
| Normal (18.5-22.9) | 25,817(55) | 20,997(54) |  |
| At risk (23.0-24.9) | 6,588(14) | 6,381(16) |  |
| Obese I (25.00-29.9) | 5,830(12) | 5,489(14) |  |
| Obese II (>30.0) | 1,270(3) | 971(3) |  |
| **Residence** |  |  | <0.001 |
| Rural | 22,423(47) | 19,326(50) |  |
| Urban | 25,295(53) | 19,469(50) |  |
| **Education** |  |  | <0.001 |
| Junior high school | 1,920(4) | 1,119(3) |  |
| High school | 23,728(49) | 15,687(40) |  |
| Diploma/certificate | 12,890(27) | 10,578(27) |  |
| University | 9,439(20) | 11,547(30) |  |
| **Income (Baht/month)** |  |  | <0.001 |
| <10,000 | 32,794(70) | 22,641(59) |  |
| 10,001-20,000 | 9,759(21) | 10,811(28) |  |
| >20,001 | 4,116(9) | 4,840(13) |  |
| **Smoking** |  |  | <0.001 |
| Never smoked | 32,812(72) | 28,313(76) |  |
| Ex-smoker | 7,373(16) | 6,036(16) |  |
| Current smoker | 5,410(12) | 3,129(8) |  |
| **Alcohol intake** |  |  | <0.001 |
| Never | 11,984(25) | 10,728(28) |  |
| Used to drink (quit) | 4,519(10) | 3,205(8) |  |
| Occasional/Social | 28,587(60) | 22,785(59) |  |
| Regular drinker | 2,289(5) | 1,882(5) |  |

*Numbers may not add to total sample size due to missing responses for some characteristics

**χ2 comparing each baseline characteristic by participation status

† Body mass Index (BMI) in kg/m^2^, categorized by Asian cut-offs using the WHO International Obesity Taskforce recommendations
